# Leukocyte TLR5 deficiency inhibits atherosclerosis by reduced macrophage recruitment and defective T-cell responsiveness

**DOI:** 10.1038/srep42688

**Published:** 2017-02-16

**Authors:** Guilielmus H.J.M. Ellenbroek, Gijs H.M. van Puijvelde, Adam A. Anas, Martine Bot, Miriam Asbach, Arjan Schoneveld, Peter J. van Santbrink, Amanda C. Foks, Leo Timmers, Pieter A. Doevendans, Gerard Pasterkamp, Imo E. Hoefer, Tom van der Poll, Johan Kuiper, Saskia C.A. de Jager

**Affiliations:** 1 Laboratory of Experimental Cardiology, University Medical Center Utrecht, The Netherlands; 2 Division of Biopharmaceutics, Leiden Academic Center for Drug Research, The Netherlands; 3 Division of Infectious Diseases, Academic Medical Center, University of Amsterdam, the Netherlands; 4 Center for Experimental and Molecular Medicine, Academic Medical Center, University of Amsterdam, The Netherlands; 5 Department of Cardiology, University Medical Center Utrecht, The Netherlands; 6 Laboratory of Clinical Chemistry and Hematology, University Medical Center Utrecht, The Netherlands

## Abstract

Toll-like receptors (TLR) provide a critical link between innate and adaptive immunity, both important players in atherosclerosis. Since evidence for the role of TLR5 is lacking, we aimed to establish this in the immune axis of atherosclerosis. We assessed the effect of the TLR5-specific ligand Flagellin on macrophage maturation and T-cell polarisation. Next, we generated TLR5^−/−^LDLr^−/−^ chimeras to study the effect of hematopoietic TLR5 deficiency on atherosclerosis formation. Flagellin stimulation did not influence wildtype or TLR5^−/−^ macrophage maturation. Only in wildtype macrophages, Flagellin exposure increased MCP-1 and IL6 expression. Flagellin alone reduced T-helper 1 proliferation, which was completely overruled in the presence of T-cell receptor activation. *In vivo*, hematopoietic TLR5 deficiency attenuated atherosclerotic lesion formation by ≈25% (1030*10^3^ ± 63*10^3^ vs. 792*10^3^ ± 61*10^3^ μm^2^; p = 0.013) and decreased macrophage area (81.3 ± 12.0 vs. 44.2 ± 6.6 μm^2^; p = 0.011). In TLR5^−/−^ chimeric mice, we observed lower IL6 plasma levels (36.4 ± 5.6 vs. 15.1 ± 2.2 pg/mL; p = 0.003), lower (activated) splenic CD4^+^ T-cell content (32.3 ± 2.1 vs. 21.0 ± 1.2%; p = 0.0018), accompanied by impaired T-cell proliferative responses. In conclusion, hematopoietic TLR5 deficiency inhibits atherosclerotic lesion formation by attenuated macrophage accumulation and defective T-cell responsiveness.

Atherosclerosis is a progressive multi-factorial disease affecting middle-sized and large arteries. It is the main culprit in the development of ischemic heart and cerebrovascular disease, together responsible for 29% of all deaths worldwide in 2013^1^. Although initially thought to be exclusively caused by lipids and risk factors such as diabetes, hypertension and smoking^2^, it is now generally acknowledged that inflammation plays a critical role in atherosclerosis^3^,^4^. Migration of leukocytes into the vessel wall is an essential step in atherosclerotic lesion initiation and progression^5^,^6^. Hence, inhibition or prevention of leukocyte recruitment towards the vessel wall may prevent or reduce plaque development. In addition to the recruitment of leukocytes, it has been shown that immune cell activation plays an important role in lesion development^7^. Best known leukocyte activators are the highly conserved toll-like receptors (TLR), which are expressed on both immune and non-immune cells^8^. Several TLR subfamilies have been linked to atherosclerotic lesion initiation and progression, of which TLR2 and TLR4 have been the most extensively studied^9^. Although TLR5 has been linked to inflammation in a variety of inflammatory diseases including atherosclerosis^10^,^11^,^12^,^13^, the role of hematopoietic TLR5 deficiency in atherosclerotic plaque formation remains unclear.

TLR5 is an extracellular receptor for bacterial Flagellin and ubiquitously expressed in almost all tissue types^14^. In addition to one or more exogenous stimuli, most TLRs also respond to specific endogenous ligands^15^. Whereas a broad variety of endogenous ligands have been described for most TLRs^9^, an equivalent for TLR5 is lacking. Since many exogenous TLR ligands are expressed in (human) atherosclerotic lesions^16^, Flagellin may also have a role in the development of atherosclerosis in the current study^17^. However, since TLR5 is an important contributor in diseases characterized by sterile inflammation (like cardiac ischemia-reperfusion injury^11^ and rheumatoid artritis^18^), it is to be expected that both endogenous as well as exogenous ligands can lead to TLR5-dependent inflammation.

In addition to specific tissue cells, TLR5 is also present on different immune cells^19^,^20^,^21^, of which macrophages and T-cells are of main importance in the context of atherosclerosis. Upon activation of the TLR5 receptor, MyD88 recruitment and activation of different intracellular kinases eventually leads to nuclear localization of NF-κB, resulting in a pro-inflammatory respons^22^. Since imbalanced inflammation is key in the development and progression of atherosclerosis, we investigated whether monocytes and T-cells lacking TLR5 differ in migratory and inflammatory behavior and whether hematopoietic TLR5 deficiency influences atherosclerotic plaque formation *in vivo*.

## Materials & Methods

### Animals

LDLr^−/−^ mice were obtained from the local animal breeding facility, C57Bl6 mice from Charles River (Maastricht, The Netherlands) and TLR5 deficient (TLR5^−/−^) mice from Oriental Bioservices (Kyoto, Japan). Mice were maintained on sterilized regular chow (RM3; Special Diet Services, Essex, U.K.), drinking water was supplied ad libitum.

A blinded observer performed data acquisition and measurements. Animal experiments were performed at the animal facility of either the Gorlaeus laboratory, Leiden University or the Laboratory of Experimental Cardiology, University Medical Center Utrecht. All animal experiments were approved by the Ethical Committee on Animal Experimentation of Leiden University (Leiden, the Netherlands) or the University Medical Center Utrecht (Utrecht, the Netherlands) and conform to the ‘Guide for the care and use of laboratory animals’.

### Bone marrow transplantation

To induce bone marrow aplasia, female LDLr^−/−^ recipient mice were exposed to a single dose of 9 Gy (0.19 Gy/min, 200 kV, 4 mA) total body irradiation using an Andrex Smart 225 Röntgen source (YXLON International) with a 6 mm aluminum filter. The day after, donor bone marrow was isolated from male WT and TLR5^−/−^ littermates by flushing the femurs and tibias. Irradiated female recipients received 0.5 × 10^7^ bone marrow cells by tail vein injection. Drinking water was supplied ad libitum and supplemented with antibiotics (83 mg/L ciprofloxacin and 67 mg/L polymyxin B sulfate and 6.5 g/L sucrose) for 14 days, after which repopulation of the bone marrow has been completed.

After a recovery period of 6 weeks, animals were placed on a Western-type diet containing 0.25% cholesterol and 15% cacao butter (SDS) for 12 weeks, after which they were sacrificed.

### Stimulation of bone marrow derived macrophages

Bone marrow cells were isolated from the tibias and femurs of male TLR5^−/−^ and C57Bl/6 mice. To obtain bone marrow-derived macrophages, cells were cultured for 7 days in RPMI supplemented with 10% FCS, 100 U/mL penicillin/streptomycin, 0.1 mM nonessential amino acids, 1% pyruvate and 2 mM L-glutamine (Thermo Fisher Scientific) in the presence of 10 ng/mL macrophage colony-stimulating factor.

For *in vitro* stimulation, 0.5 × 10^6^ macrophages were added per well (24-well cell culture plates (Greiner Bio-One)). Cells were stimulated with 1 ng/mL Flagellin Ultrapure (Invivogen, Toulouse, France) or 1 ng/mL LPS (Sigma Aldrich, Zwijndrecht, the Netherlands) as a positive control. After 24 hours, cells were harvested for RNA isolation.

### RT-PCR

Total RNA was isolated from the stimulated WT and TLR5^−/−^ macrophages using Trizol reagent according to manufacturer’s instructions (Invitrogen, Breda, the Netherlands). RNA was reverse transcribed using M-MuLV reverse transcriptase (RevertAid, MBI Fermentas, Leon-Roth). The expression levels of inducible nitric oxide synthase (iNOS), Arginase-1, monocyte chemoattractant protein-1 (MCP-1), C-C chemokine receptor type 2 (CCR2), and interleukin 6 (IL6) were analyzed by real time polymerase chain reaction (RT-PCR, Taqman, Applied Bioscience). Primer sequences are depicted in Table 1. The mRNA expression was determined relative to the average expression of three household genes: acidic ribosomal phosphoprotein PO (36B4), hypoxanthine phosphoribosyltransferase (HPRT) and 60S ribosomal protein L27 (Rpl27).

### Luminex

Circulating levels of IL6, IL17A and interferon-γ (IFN-γ) were determined by luminex according to the manufacturers protocol. A multiplex panel (eBioscience Mouse Th1/Th2 essential 6-plex and Mouse IL17A Simplex) was used in combination with the “Bio-Plex Multiplex system (Bio-Rad)” to perform luminex analyses.

### *Ex vivo* proliferation and T-cell polarisation

Spleens were isolated from WT and TLR5^−/−^ chimeras to assess *ex vivo* proliferation or C57Bl/6 WT mice to assess T-cell polarization. Spleens were gently squeezed through a 70 μm mesh cell strainer (Becton Dickinson, San Diego, CA, USA) to obtain a single cell suspension. Cells were washed and resuspended in RPMI1640 (supplemented with 10% FCS, 20 mM L-glutamine, 100 U/ml penicillin and 100 μg/mL streptomycin) and seeded at a density of 3 × 10^5^ cells/well in a 96 well u-bottom cell culture plate (Greiner Bio One, Alphen aan den Rijn, the Netherlands). Cells were stimulated for 72 hours with medium alone, 1 ng/mL Flagellin Ultrapure, 1 ng/mL Flagellin Ultrapure in combination with 2 μg/mL anti-CD3 (eBioscience) or 2 μg/mL anti-CD3 in combination with 2 μg/mL anti-CD28 as a positive control. To assess proliferation, cells were incubated with 0.5 μCi [3 H]Thymidine during the last 16 hours of 3 days in culture. To quantify thymidine incorporation the cells were washed with PBS and lysed with 0.1 M NaOH and cell-associated radioactivity was determined by liquid scintillation counting. To assess T-cell polarisation, after 72 hours of stimulation, 10 μg/mL Brefeldin-A was added overnight to retain the proteins inside the cell. The next morning cells were harvested for flow cytometric analysis.

### Intracellular flow cytometry

Cultured T-cells were harvested, centrifuged and resuspended for intracellular staining according to the manufacturers protocol (eBioscience). In short, cells were resuspended in PBS supplemented with 1% normal mouse serum and stained for the cell surface marker CD4 (BD Biosciences) for 30 minutes in the dark at 4 °C. Cells were washed to remove unbound antibody and resuspended in IC fixation buffer. After 30 minutes, permeabilisation buffer was added, cells were centrifuged (1500 rpm, 5 min) and resuspended in permeabilisation buffer. Antibodies for intracellular antigens Tbet, IFN-γ, RORγt, IL17A and Foxp3 (eBioscience) were added and incubated for 45 minutes at room temperature in the dark. Cells were washed twice in permeabilisation buffer and resuspended in flow cytometry staining buffer for cell acquisition (FACSCanto, BD Biosciences).

### Flow cytometry

Blood, spleen and bone marrow cells were isolated from WT and TLR5^−/−^ mice that had been fed a Western-type diet for 1 week. Bone marrow was isolated by flushing the femurs and tibias of WT and TLR5^−/−^ mice with PBS. Subsequently, the cell suspension was gently filtered through a 70 μm cell strainer to obtain a single cell suspension (70 μm pores, BD Bioscience). Spleens were harvested and single-cell suspensions of splenocytes were prepared by gently mincing the spleen through a cell strainer (70 μm pores, BD Bioscience). Bone marrow cells and splenocytes were incubated at 4 °C with erythrocyte lysis buffer (155 mM NH4CL in 10 mM Tris/HCL, pH 7.2) for 5 minutes. Cells were centrifuged for 5 minutes at 1500 rpm and then resuspended in lysis buffer to remove residual erythrocytes. Cells were washed twice with PBS. 50 μL whole blood, bone marrow (300.000 cells), or spleen cell suspensions (300.000 cells) were stained for the surface markers CD11B (eBioscience), Ly6G (1A8) (Biolegend), Ly6C (Biolegend) and CCR2 (R&D Systems) and incubated for 30 minutes in the dark. Subsequently cells were either washed in PBS and subjected to flow cytometric analyses (Gallios, Beckman Coulter) or prepared for intracellular staining.

For intracellular assessment of cytokine content, cells were washed to remove unbound antibody and resuspended in IC fixation buffer (4° Celsius). After 60 minutes, cells were centrifuged (1500 rpm, 5 min) and resuspended in permeabilisation buffer, washed once more and resuspended in permeabilisation buffer. Antibodies for intracellular antigens IL10 and IL12 (eBioscience, San Diego, CA, USA) were added and incubated for 30 minutes at room temperature in the dark. Cells were washed twice in permeabilisation buffer and resuspended in flow cytometry staining buffer for cell acquisition (Gallios, Beckman Coulter). FACS data were analyzed with Kaluza software (Beckman Coulter) and gated according to the strategy depicted in supplemental Fig. 1.

### Histological analyses

Cryosections of the aortic root (10 μm) were collected and stained with Oil-red-O for morphometric analyses. Corresponding sections on separate slides were used for immunohistochemical stainings. Macrophages were stained with an antibody directed against MOMA-2 (monoclonal rat IgG2b, dilution 1:50; Serotec, Oxford, United Kingdom). Goat anti-rat IgG-AP (dilution 1:100; Sigma, St. Louis, MO) was used as a secondary antibody and NBT-BCIP (Dako, Glostrup, Denmark) as an enzyme substrate. CD4^+^ T-cells were stained using CD4 (monoclonal rabbit, dilution 1:50; Cell Marque, Darmstadt, Germany). BrightVision poly-AP anti-rabbit (Immunologic, Duiven, The Netherlands) was used as a secondary antibody and liquid permanent red (DAKO) as an enzyme substrate. CD8^+^ T-cells were stained using CD8a (monoclonal rat IgG2a,λ, dilution 1:50, eBioscience). The ImmPRESS HRP anti-Rat IgG, Mouse adsorbed (Peroxidase) Polymer Detection Kit (Vector, Burlingame, CA, USA) was used as a secondary antibody and DAB (Sigma, St. Louis, MO, USA) as an enzyme substrate. Collagen was visualized by a Masson’s trichrome staining (Sigma). The section with the largest lesion and at least four flanking sections were used to determine lesion size. At least two flanking sections were used to analyse macrophage, T-cell and collagen content. The percentage of macrophages and collagen in the lesions was determined by dividing the area positive for MOMA-2 or collagen by the total lesion surface area. Necrotic core size was determined in the Oil-red-O stained sections and defined as an a-cellular, lipid-poor region. CD4^+^ and CD8^+^ T-cells were manually counted and expressed as counts/mm^2^.

Histological analyses were performed in a blinded fashion using a Leica DMRE Microscope equipped with a Leica DC500 camera and Qwin quantification software (Leica, Rijswijk, the Netherlands). Histological analyses of CD4^+^ and CD8^+^ T-cell quantification were performed on an Olympus BX53 microscope equipped with a DP71 camera and CellSens quantification software (Olympus Corporation, Tokyo, Japan).

### Statistical analysis

Data are expressed as mean ± standard error of the mean (SEM). Data distribution was evaluated using a Shapiro-Wilk test. A two-tailed student’s t-test was used to compare individual groups if data were normally distributed, whereas non-parametric data were analyzed using a Mann-Whitney U test. Multiple groups were compared by one-way ANOVA with a post-hoc two-tailed student’s t-test between significantly different groups. A level of p < 0.05 was considered significant. Statistical analyses were performed using GraphPad Prism 6.

## Results

### Absence of TLR5 affects migratory potential of macrophages *in vitro*

**Figure 1:**
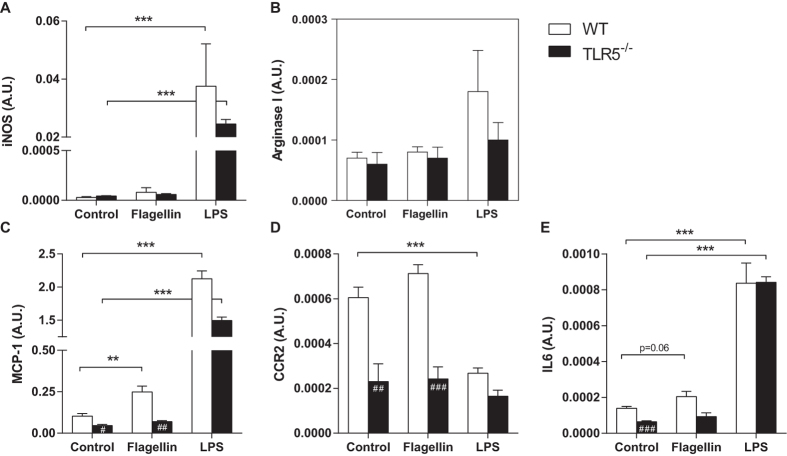
*In vitro* polarisation and migratory potential of macrophages. Stimulation of WT and TLR5^−/−^ MCSF-derived macrophages with Flagellin or LPS did not induce significant differences in iNOS (**A**) and Arg-1 (**B**) gene expression between both groups. Already at baseline, WT macrophages showed higher mRNA levels of MCP-1 (**C**), CCR2 (**D**) and IL6 (**E**), of which the expression of MCP-1 and IL6 increased upon Flagellin stimulation. No such increase was observed in TLR5^−/−^ macrophages upon stimulation with Flagellin. The expression of CCR2 was also different at baseline between both groups, but no change in expression level was observed after stimulation with Flagellin (**D**); n = 4 per group. *WT: wildtype, TLR5*^−/−^*: toll-like receptor 5 knockout, #p* < *0.05, ##p* < *0.01, ###p* < 0.001, **p < 0.01, ***p < *0.001*.

To study the effect of TLR5 signaling on immune cells, bone marrow derived macrophages from WT and TLR5^−/−^ mice were stimulated *ex vivo* with the TLR5 ligand Flagellin. Cells stimulated with LPS served as a positive control. As measured by iNOS and Arginase I expression, no preferential skewing towards either M1 (8.0*10^−5^ ± 4.7*10^−5^ vs. 6.0*10^−5^ ± 7.3*10^−6^; p = 0.69, Fig. 1A) or M2 (8.0*10^−5^ ± 8.9*10^−6^ vs. 7.0*10^−5^ ± 1.8*10^−5^; p = 0.32, Fig. 1B) phenotype was observed upon stimulation with Flagellin. Already at baseline, TLR5^−/−^ macrophages showed a lower expression of the potent monocyte chemoattractant MCP-1 (0.103 ± 0.015 in WT vs. 0.046 ± 0.014 A.U. in TLR5^−/−^; p = 0.013, Fig. 1C). Upon stimulation with Flagellin, the expression of MCP-1 increased only in WT macrophages (0.249 ± 0.071 in WT vs. 0.070 ± 0.013 A.U. in TLR5^−/−^; p = 0.0025). The expression of the MCP-1 receptor CCR2 was also lower in TLR5^−/−^ macrophages at baseline (6.0*10^−4^ ± 4.8*10^−5^ in WT vs. 2.3*10^−4^ ± 7.8*10^−5^ A.U. in TLR5^−/−^; p = 0.0069, Fig. 1D), but showed no further increase upon exposure to Flagellin in either WT (6.0*10^−4^ ± 4.8*10^−5^ vs. 7.1*10^−4^ ± 3.9*10^−5^ A.U.; p = 0.13) or TLR5^−/−^ macrophages (2.3*10^−4^ ± 7.8*10^−5^ vs. 2.4*10^−4^ ± 5.3*10^−5^ A.U.; p = 0.92). In addition, expression levels of IL6 were significantly higher in WT macrophages at baseline (1.4*10^−4^ ± 9.5*10^−6^ in WT vs. 6.0*10^−5^ ± 5.1*10^−6^ A.U. in TLR5^−/−^; p = 0.0003, Fig. 1E) and did marginally increase upon Flagellin stimulation in WT macrophages (1.4*10^−4^ ± 9.5*10^−6^ vs. 2.1*10^−4^ ± 2.9*10^−5^ A.U.; p = 0.06).

### *In vitro* TLR5 stimulation induces regulatory T-cell differentiation

**Figure 2:**
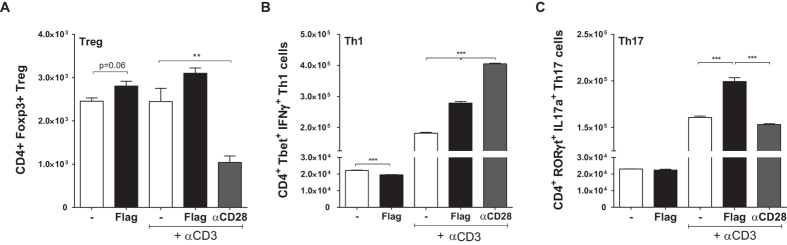
*In vitro* polarisation of T-cells. Exposure of Flagellin to WT CD4^+^ T-cells either in the presence or absence of αCD3 resulted in a mild but insignificant induction in the number of Tregs (**A**). The number of Th1-cells decreased upon Flagellin stimulation only (**B**), whereas no change was observed in Th17-cells (**C**). In the presence of both Flagellin and αCD3, the induction of Th1- and Th17-cells robustly increased (**B**,**C**); n = 3 per group. WT: wildtype, Tregs: regulatory T-cells, Th1-cells: T helper type 1 cells, Th17: T helper type 17 cells, **p < 0.01, ***p < *0.001*.

In addition to cells of the myeloid lineage, we observed expression of TLR5 in ≈14% of CD4^+^ T-cells, 44% of activated T-cells and 60% of the regulatory T-cells (data not shown). The effect of TLR5 signaling on CD4^+^ T-cell polarisation was studied by stimulating C57Bl/6 derived T-cells with Flagellin in the absence or presence of αCD3. Stimulation with a combination of αCD3 and αCD28 served as a positive control. The number of regulatory T-cells was mildly induced upon exposure to Flagellin alone (2455 ± 74 vs. 2807 ± 109; p = 0.056, Fig. 2A) or in combination with αCD3 (2448 ± 306 vs. 3100 ± 124; p = 0.14). The number of T-helper 1 (Th1) cells decreased upon Flagellin stimulation alone (22196 ± 203 vs. 19552 ± 150; p = 0.0005), Fig. 2B), but increased when exposed to a combination of Flagellin and αCD3 (181764 ± 2777 vs. 279000 ± 4879; p < 0.0001). Exposing CD4^+^ T-cells to Flagellin alone did not affect the number of T-helper 17 (Th17) cells (23064 ± 86 vs. 22385 ± 522; p = 0.27, Fig. 2C), but when exposed to a combination of Flagellin and αCD3 the number of Th17-cells robustly increased (160854 ± 1439 vs. 199392 ± 4142; p = 0.0001).

### Hematopoietic TLR5 deficiency reduces plaque size, macrophage area and necrotic core in LDLr^−/−^ mice

**Figure 3:**
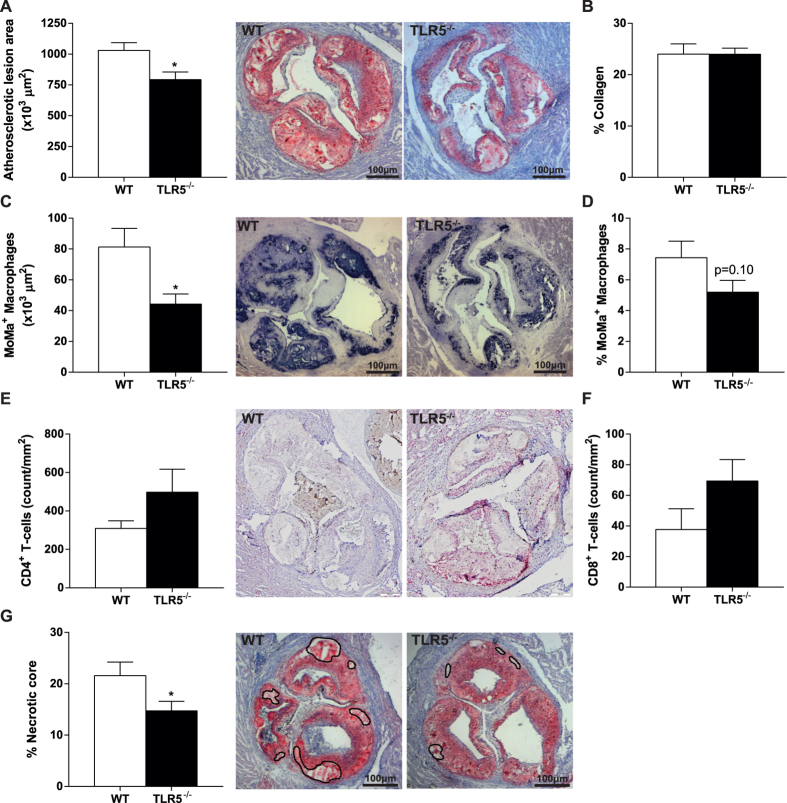
Atherosclerotic plaque size and composition. After 12 weeks of high-fat diet, the atherosclerotic plaque area was significantly larger in WT mice compared to TLR5^−/−^ mice (**A**). No difference between both groups was observed with respect to collagen content (**B**), yet the plaque of TLR5^−/−^ mice showed a decrease in macrophage influx (**C**,**D**). Although not significant, TLR5^−/−^ mice showed higher numbers of CD4^+^ (**E)** and CD8^+^ T-cells (**F**). The necrotic core expressed as a percentage of the plaque was significantly higher in WT mice (**G**); n = 12–13 per group (5-8 for CD4-8 staining). *WT: wildtype, TLR5*^−/−^: toll-like receptor 5 knockout, *p < *0.05*.

To assess the effect of hematopoietic TLR5 deficiency on atherosclerotic lesion development, TLR5^−/−^ bone marrow chimeras were generated. Plasma cholesterol and body weight were monitored during the experiment and did not significantly differ between WT and TLR5^−/−^ chimeras (data not shown). After 12 weeks of high fat diet feeding, mice were sacrificed and atherosclerotic lesion size in the aortic root was analyzed. In TLR5^−/−^ chimeras, lesion size was markedly reduced by ≈25% (1030*10^3^ ± 63*10^3^ in WT vs. 792*10^3^ ± 61*10^3^ μm^2^ in TLR5^−/−^; p = 0.013, Fig. 3A), whereas collagen content as a percentage of total plaque area was not different between both groups (24.0 ± 2.0 in WT vs. 24.0 ± 1.2% in TLR5^−/−^; p = 0.99, Fig. 3B). The decrease in lesion size was accompanied by a reduction in macrophage area (81.3 ± 12.0 in WT vs. 44.2 ± 6.6 μm^2^ in TLR5^−/−^; p = 0.011, Fig. 3C), however lesions in TLR5^-/-^ chimeras did not show a difference with respect to the percentage of macrophage area (7.4 ± 1.1 in WT vs. 5.2 ± 0.8% in TLR5^−/−^; p = 0.10, Fig. 3D). Despite the observed decrease in splenic T-cell content we did not observe a difference in the number of CD4^+^ T-cells accumulated in the plaques of TLR5^−/−^ mice (309 ± 89 in WT vs. 498 ± 338/mm2 in TLR5^−/−^; p = 0.17, Fig. 3E). Similarly, no difference was observed in the number of CD8^+^ T-cells (38 ± 30 in WT vs. 69 ± 40/mm2 in TLR5^−/−^; p = 0.16, Fig. 3F). In agreement with smaller lesion size, necrotic core size was reduced (21.6 ± 2.7 in WT vs. 14.7 ± 1.9% in TLR5^−/−^; p = 0.046, Fig. 3G).

### The numbers of circulating monocytes, T-cells and their subsets are influenced by TLR5 deficiency

One week after feeding WT and TLR5^−/−^ mice a Western-type diet, flow cytometry was used to assess several monocyte subsets and receptor expression on blood and bone marrow. We observed that TLR5^-/-^ mice displayed a decreased number of circulating pro-inflammatory Ly6C^high^ monocytes (498 ± 75 in WT vs. 367 ± 57 cells/uL in TLR5^−/−^; p = 0.01, Table 2). In addition, TLR5^−/−^ circulating monocytes showed a decrease in pro-inflammatory IL12 staining (140 ± 55 in WT vs. 38 ± 8 cells/uL in TLR5^−/−^; p = 0.003, Table 2). In the bone marrow, we observed a decrease in the number of monocytes as a percentage of all viable cells (11.4 ± 0.5 in WT vs. 9.1 ± 0.5% in TLR5^−/−^; p < 0.0001, Table 2) and specifically, a decrease in Ly6C^high^ monocytes (10.1 ± 0.7 in WT vs. 7.7 ± 0.4% in TLR5^−/−^; p = 0.003, Table 2). Both, blood and bone marrow showed a decrease in the percentage of CCR2^+^ cells.

**Figure 4:**
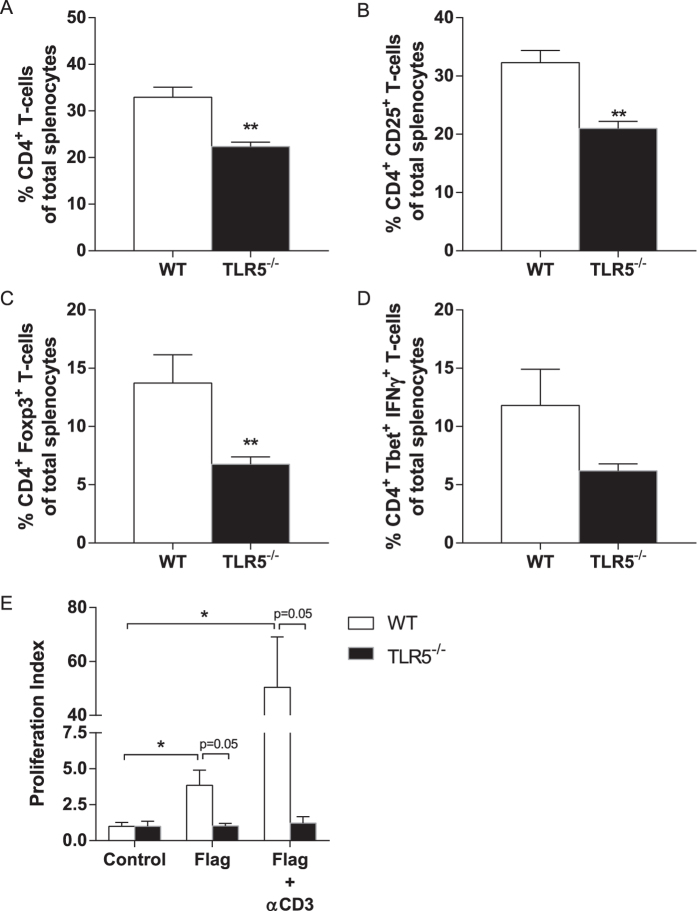
Splenic T-cell numbers, subsets and proliferation indices. The number of CD4^+^ T-cells as a percentage of total splenocytes was higher in WT mice compared to TLR5^−/−^ mice (**A**), similar to the percentage of activated CD4^+^ CD25^+^ T-cells (**B**). Tregs expressed as a percentage of total splenocytes were significantly higher in WT mice (**C**) and, yet insignificantly, so was the percentage of Th1-cells (**D**). T-cells isolated from the spleen were used for proliferation indices in both groups (**E**). No differences were observed at baseline. Upon stimulation with Flagellin alone the proliferation index was increased in only the WT T-cells, an effect that was even more pronounced in the presence of αCD3. No such differences were observed with Flagellin either in the presence or absence of αCD3 with the TLR5^−/−^ T-cells; n = 4–6 per group. *TLR5*^−/−^: toll-like receptor 5 knockout, WT: wildtype, Tregs: regulatory T-cells, Th1-cells: T helper type 1 cells, *p < 0.05, **p < *0.01*.

T-cell subsets were determined in the hematopoietic TLR5^−/−^ mice at the end of the experiment. The number of CD4^+^ T-cells as a percentage of total splenocytes was lower in TLR5^−/−^ mice (32.9 ± 2.2 in WT vs. 22.4 ± 1.0% in TLR5^−/−^; p = 0.0013, Fig. 4A), as was the percentage of activated CD4^+^ CD25^+^ T-cells (32.3 ± 2.1 in WT vs. 21.0 ± 1.2% in TLR5^−/−^; p = 0.0018, Fig. 4B). Similarly, TLR5^−/−^ mice displayed lower percentages of Tregs (13.7 ± 2.4 in WT vs. 6.8 ± 0.6% in TLR5^−/−^; p = 0.0087; Fig. 4C) and a slightly lower percentage of Th1-cells (11.8 ± 3.1 in WT vs. 6.2 ± 0.6% in TLR5^−/−^; p = 0.13, Fig. 4D).

*Ex vivo* T-cell proliferation was performed to assess complete replacement of T-cells upon bone marrow transplantation (Fig. 4E). Proliferation under control, non-stimulatory conditions did not differ between WT and TLR5^−/−^ cells (p = 1.00). Proliferation upon stimulation with Flagellin alone or Flagellin in combination with αCD3 is displayed as fold induction to control proliferation. Stimulation with Flagellin led to an increased proliferation index in WT mice (3.8 ± 1.1 upon stimulation vs. 1.0 ± 0.3 in non-stimulatory conditions; p = 0.03), but did not in TLR5^−/−^ mice (p = 0.96). In the presence of both Flagellin and αCD3, T-cell proliferation was robustly induced in WT (50.4 ± 18.7 upon stimulation vs. 1.0 ± 0.3; p = 0.03), but not in TLR5^−/−^ mice (1.2 ± 0.4 upon stimulation vs. 1.0 ± 0.3; p = 0.71). Flagellin-independent proliferation was normal in TLR5^−/−^ T-cells as shown by αCD3/αCD28-induced proliferation (proliferation index 85.3 ± 55 in TLR5^−/−^ and 96.7 ± 23 in WT, data not shown).

### Hematopoietic TLR5 deficiency influences pro-inflammatory cytokine levels

**Figure 5:**
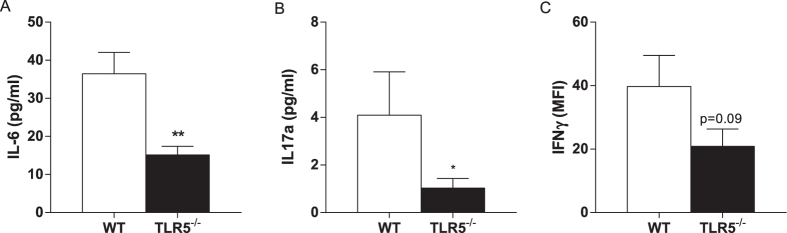
Plasma levels of pro-inflammatory cytokines. Plasma levels of IL6 (**A**) and IL17A (**B**) determined by luminex were significantly lower in TLR5^−/−^ mice. Yet insignificant, also the plasma levels of IFN-γ were lower in TLR5^−/−^ compared to WT mice (**C**); n = 8–10 mice per group. *TLR5*^−/−^: toll-like receptor 5 knockout, WT: wildtype, IL: interleukin, IFN-γ: interferon γ, *p < 0.05, **p < *0.01*.

Luminex was used for the quantification of pro-inflammatory cytokine levels in plasma of WT and TLR5^−/−^ mice at sacrifice. Compared to WT animals, significantly lower levels of IL6 (36.4 ± 5.6 in WT vs. 15.1 ± 2.2 pg/mL in TLR5^−/−^; p = 0.0029, Fig. 5A) and IL17A (4.1 ± 1.8 in WT vs. 1.0 ± 0.4 pg/mL in TLR5^−/−^; p = 0.028, Fig. 5B) were detected in plasma of TLR5^−/−^ mice. Plasma IFN-γ levels were relatively low and did not allow us to reliably calculate the concentration. Therefore, we used mean fluorescence intensity (MFI) to compare both groups, which showed slightly decreased IFN-γ levels in TLR5^−/−^ mice compared WT mice (39.7 ± 9.8 in WT vs. 20.9 ± 5.4 in TLR5^−/−^ MFI; p = 0.09, Fig. 5C).

## Discussion

Over the past decades there has been a widespread interest in the role of TLRs in the onset and progression of cardiovascular diseases. In atherosclerosis, TLR2 and TLR4 have been the most prominently studied^9^ and evidence for the role of TLR5 has been previously shown^12^,^13^. In this regard, the current paper is the first to show that hematopoietic TLR5 deficiency attenuates atherosclerosis formation in LDLr^−/−^ mice. In addition, the plaques of these mice contain less macrophages and a smaller necrotic core compared to mice that received WT bone marrow. These findings are in line with previous studies that show the effect of TLR5 in atherosclerotic plaque formation and inflammatory cell accumulation^12^,^13^. It also confirms the observation that inflammation – and TLRs in particular – are key drivers of atherosclerosis^3^,^9^,^12^.

In general, lack of TLRs is believed to decrease atherosclerotic burden through the impairment of pro-inflammatory signaling. In this respect, macrophages from WT mice showed an increased expression of MCP-1 and IL6 upon Flagellin stimulation, whereas no difference was observed upon stimulation of TLR5 deficient macrophages. Also, TLR5^−/−^ mice showed a decrease in circulating and bone-marrow derived Ly6C^high^ monocytes and a decrease in pro-inflammatory cytokine production (IL10, IL12). Correspondingly, plasma levels of several pro-inflammatory cytokines were higher in WT mice than in TLR5^−/−^ bone marrow chimeras.

The fact that we observe a decrease in the Ly6C^high^ monocyte pool in both the bone marrow and circulation suggests that monocyte recruitment to the plaque may be impaired. In addition, CCR2 expression on the bone marrow monocyte pool is significantly decreased in TLR5 deficient mice, which partly reflects previous research showing that CCR2 deficiency attenuates macrophage influx into the plaque^23^. Decreased macrophage CCR2 expression could have had a similar effect in the current manuscript. In addition to the impairment in pro-inflammatory signaling, this may very well be one of the primary mechanisms in the reduction of atherosclerotic plaque formation. These findings are in accordance with previous studies showing that TLR5 stimulation of mouse endothelial cells enhanced the expression of pro-inflammatory molecules and increased the adhesive and migratory capacity for monocytes^12^. In addition, TLR5 inhibition showed decreased serum levels of several pro-inflammatory cytokines^13^. Similarly, ApoE^−/−^TLR4^−/−^ mice on high-cholesterol feeding showed a reduction in serum MCP-1 levels, plaque size and macrophage infiltration when compared to ApoE^−/−^ mice^24^. Again, this emphasizes the importance of migratory proteins (MCP-1, IL6, etc.) in atherogenesis and inflammatory cell deposition^5^,^6^,^23^,^25^.

In addition to TLR5 being abundantly expressed on cells of myeloid origin, we observed expression of TLR5 on CD4^+^ T-cells as well. When exposing T-cells to Flagellin in a non-inflammatory condition, an increase in the number of Tregs and a decrease in Th1-cells were observed. These data are similar to a previous study showing that TLR5 stimulation enhances the suppressive capacity of regulatory T-cells^26^. However, when performing these experiments under more inflammatory conditions by adding αCD3 to simulate antigen-T-cell receptor interaction, the suppressive effect of Tregs was lost and actually proliferation of pro-atherogenic Th1- and Th17-cells was stimulated. This implicates a pro-atherogenic effect of stimulation of TLR5 on T-cells in a pro-inflammatory environment, like observed in atherosclerosis. Indeed, this was reflected *in vivo* by lower numbers of Tregs, activated CD4^+^ CD25^+^ cells and Th1-cells and decreased IL6, IL17A and IFN-γ plasma levels in TLR5^−/−^ bone marrow chimeras. Despite this general effect on CD4^+^ T-cells, no significant reduction in the number of CD4^+^ or CD8^+^ T-cells in the atherosclerotic plaque was observed.

Apart from their importance as pattern recognition receptors (PRR) of exogenous (mostly bacterial) ligands, TLRs can also potently respond to specific disease-associated endogenous ligands^15^. Whereas a broad variety of endogenous ligands have been described for several TLRs^9^, an equivalent for TLR5 is lacking. Since many exogenous TLR ligands are expressed in (human) atherosclerotic lesions^16^, Flagellin may indeed be (partially) responsible for the results observed in the current study, implicating a role of (subclinical) infection in the development of atherosclerosis^17^. However, this phenomenon may be debated on by accumulating evidence showing that TLR5 also plays an important role in diseases characterized by sterile inflammation, such as cardiac ischemia-reperfusion injury^11^ and rheumatoid artritis^18^.

Although it remains unclear which ligand is responsible for the observed effect in the current study, we do show the importance of hematopoietic TLR5 deficiency in the early stage of atherosclerosis formation. Whether this also holds true for a model of atherosclerosis with larger and more complex lesions remains to be elucidated. In this regard, hematopoietic TLR2 deficiency has also been shown to be important in the early stage of atherosclerosis formation, but was not evident when feeding mice a 1.5% instead of a 0.15% cholesterol-enriched diet^27^. Moreover, although baseline TLR5 deficiency was able to attenuate atherosclerotic plaque formation, from a clinical perspective it remains of interest to see whether TLR5 inhibition at a later stage would also be able to reduce or stabilize already manifest atherosclerosis.

## Conclusion

In conclusion, the present manuscript is one of the first to show that hematopoietic TLR5 deficiency in mice attenuates atherosclerotic plaque development through decreased macrophage accumulation and defective T-cell responsiveness. Still, more knowledge has to be gathered on the possibility to antagonize TLR5 signaling to reduce or stabilize previously established atherosclerotic lesions.

## Additional Information

**How to cite this article**: Ellenbroek, G. H.J.M. *et al*. Leukocyte TLR5 deficiency inhibits atherosclerosis by reduced macrophage recruitment and defective T-cell responsiveness. *Sci. Rep.* **7**, 42688; doi: 10.1038/srep42688 (2017).

**Publisher's note:** Springer Nature remains neutral with regard to jurisdictional claims in published maps and institutional affiliations.

## Supplementary Material

Supplementary Figure

## Figures and Tables

**Table 1:** Primer sequences.

Gene	Forward	Reverse
iNOS	CCTGGTACGGGCATTGCT	GCTCATGCGGCCTCCTTT
Arginase-1	TGGCAGAGGTCCAGAAGAATGG	GTGAGCATCCACCCAAATGACAC
MCP-1	AGGTCCCTGTCATGCTTCTG	TCTGGACCCATTCCTTCTTG
CCR2	AGAGAGCTGCAGCAAAAAGG	GGAAAGAGGCAGTTGCAAAG
IL6	AGACAAAGCCAGAGTCCTTCAGAGA	GGAGAGCATTGGAAATTGGGGTAGG
36B4	CTGAGTACACCTTCCCACTTACTGA	CGACTCTTCCTTTGCTTCAGCTTT
HPRT	TACAGCCCCAAAATGGTTAAGG	AGTCAAGGGCATATCCAACAAC
Rpl27	CGCCAAGCGATCCAAGATCAAGTCC	AGCTGGGTCCCTGAACACATCCTTG

iNOS: inducible Nitric Oxide Synthase, MCP-1: monocyte chemoattractant protein-1, CCR2: C-C chemokine receptor type 2 (CCR2), IL6: interleukin 6, 36B4: acidic ribosomal phosphoprotein PO, HPRT: hypoxanthine phosphoribosyltransferase, Rpl27: 60S ribosomal protein L27.

**Table 2:** Monocyte subsets and their pro-inflammatory and migratory protein levels.

	Blood (cells/μl)				Bone Marrow (% of viable cells)			
**Cell (sub)type**	**Wildtype**	**TLR5**^−/−^		**p**	**Wildtype**	**TLR5**^−/−^		**p**
Monocytes	776 ± 124	621 ± 97	=	0.06	11.4 ± 0.5	9.1 ± 0.5	↓	** < 0.0001**
Ly6C^high^ mono	498 ± 75	367 ± 57	**↓**	**0.01**	10.1 ± 0.7	7.7 ± 0.4	↓	**0.0002**
Ly6C^low^ mono	291 ± 91	274 ± 49	=	0.73	1.3 ± 0.2	1.4 ± 0.1	**=**	0.54
CCR2^+^ mono	176 ± 36	154 ± 33	=	0.35	7.2 ± 0.4	5.8 ± 0.3	↓	**0.0006**
CCR2^+^ Ly6C^high^ mono	102 ± 24	90 ± 16	=	0.37	6.5 ± 0.5	5.0 ± 0.3	↓	**0.0005**
Ratio Ly6C^hi/low^	1.83 ± 0.53	1.34 ± 0.04	=	0.08	8.1 ± 1.8	5.7 ± 0.15	↓	**0.02**
IL12^+^ mono	140 ± 55	38 ± 8	**↓**	**0.003**	n.d.	n.d.		
IL10^+^ mono	307 ± 69	249 ± 51	=	0.16	n.d.	n.d.		
Ratio IL12/IL10	0.45 ± 0.10	0.16 ± 0.05	**↓**	**0.0003**	n.d.	n.d.		

Mono (=monocytes) were selected as CD11b^+^, Ly6G^−^, Ly6C^+^ cells. n.d. = not determined.

## References

[b1] GBD 2013 Mortality and Causes of Death Collaborators*. Global, regional, and national age – sex specific all-cause and cause-specific mortality for 240 causes of death, 1990–2013: a systematic analysis for the Global Burden of Disease Study 2013. Lancet 385, 117–171 (2014). 25530442 10.1016/S0140-6736(14)61682-2 PMC4340604

[b2] Kannel W. B., McGee D. & Gordon T. A General Cardiovascular Risk Profile: The Framingham Study. Am J Cardiol 36, 46–51 (1976). 10.1016/0002-9149(76)90061-8 132862

[b3] Libby P., Ridker P. M. & Maseri A. Inflammation and atherosclerosis. Circulation 105, 1135–1143 (2002). 11877368 10.1161/hc0902.104353

[b4] Hamirani Y. S. . Markers of inflammation and coronary artery calcification: a systematic review. Atherosclerosis 201, 1–7 (2008). 18561934 10.1016/j.atherosclerosis.2008.04.045

[b5] Weber C. Chemokines: Key Regulators of Mononuclear Cell Recruitment in Atherosclerotic Vascular Disease. Arterioscler Thromb Vasc Biol 24, 1997–2008 (2004). 15319268 10.1161/01.ATV.0000142812.03840.6f

[b6] Liehn E. a, Zernecke A., Postea O. & Weber C. Chemokines: inflammatory mediators of atherosclerosis. Arch Physiol Biochem 112, 229–238 (2006). 17178596 10.1080/13813450601093583

[b7] Hansson G. K. & Libby P. The immune response in atherosclerosis : a double-edged sword. Nat Rev Immunol 6, 508–519 (2006). 16778830 10.1038/nri1882

[b8] Medzhitov R. Toll-like receptors and innate immunity. Nat Rev Immunol 1, 135–145 (2001). 11905821 10.1038/35100529

[b9] Falck-hansen M. & Kassiteridi C. Toll-Like Receptors in Atherosclerosis. Int J Mol Sci 14, 14008–14023 (2013). 23880853 10.3390/ijms140714008 PMC3742229

[b10] Blohmke C. J. . TLR5 as an anti-inflammatory target and modifier gene in cystic fibrosis. J Immunol 185, 7731–8 (2010). 21068401 10.4049/jimmunol.1001513

[b11] Parapanov R. . Toll-like receptor 5 deficiency exacerbates cardiac injury and inflammation induced by myocardial ischaemia-reperfusion in the mouse. Clin Sci 129, 187–198 (2015). 25757463 10.1042/CS20140444

[b12] Kim J., Seo M., Kim S. K. & Bae Y. S. Flagellin-induced NADPH oxidase 4 activation is involved in atherosclerosis. Sci Rep 1–16, doi: 10.1038/srep25437 (2016). 27146088 PMC4857127

[b13] Zhang Y. & Zhang Y. Pterostilbene, a novel natural plant conduct, inhibits high fat-induced atherosclerosis inflammation via NF-κB signaling pathway in Toll-like receptor 5 (TLR5) deficient mice. Biomed Pharmacother 81, 345–355 (2016). 27261612 10.1016/j.biopha.2016.04.031

[b14] Zarember K. A., Godowski P. J. & Alerts E. Tissue Expression of Human Toll-Like Receptors and Differential Regulation of Toll-Like Receptor mRNAs in Leukocytes in Response to Microbes, Their Products, and Cytokines. J Immunol 168, 554–561 (2002). 11777946 10.4049/jimmunol.168.2.554

[b15] Rifkin I. R., Leadbetter E. A., Busconi L., Viglianti G. & Marshak-Rothstein A. Toll-like receptors, endogenous ligands, and systemic autoimmune disease. Immunol Rev 204, 27–42 (2005). 15790348 10.1111/j.0105-2896.2005.00239.x

[b16] Monaco C., Cole J. E. & Georgiou E. The expression and functions of toll-like receptors in atherosclerosis. Mediators Inflamm, doi: 10.1155/2010/393946 (2010). PMC2905957 20652007

[b17] Rosenfeld M. E. & Campbell L. A. Pathogens and atherosclerosis: Update on the potential contribution of multiple infectious organisms to the pathogenesis of atherosclerosis. Thromb Haemost 106, 858–867 (2011). 22012133 10.1160/TH11-06-0392

[b18] Kassem A., Henning P., Kindlund B., Lindholm C. & Lerner U. H. TLR5, a novel mediator of innate immunity-induced osteoclastogenesis and bone loss. FASEB J 29, 4449–4460 (2015). 26207027 10.1096/fj.15-272559

[b19] Gewirtz A. T. . Cutting Edge: Bacterial Flagellin Activates Basolaterally Expressed TLR5 to Induce Epithelial Proinflammatory Gene Expression. J Immunol 167, 1882–1885 (2001). 11489966 10.4049/jimmunol.167.4.1882

[b20] Hornung V. . Quantitative Expression of Toll-Like Receptor 1 − 10 mRNA in Cellular Subsets of Human Peripheral Blood Mononuclear Cells and Sensitivity to CpG Oligodeoxynucleotides. J Immunol 168, 4531–4537 (2002). 11970999 10.4049/jimmunol.168.9.4531

[b21] Okamura Y. . The Extra Domain A of Fibronectin Activates Toll-like Receptor 4*. J Biol Chem 276, 10229–10233 (2001). 11150311 10.1074/jbc.M100099200

[b22] Hayashi F. . The innate immune response to bacterial flagellin is mediated by Toll-like receptor 5. **410,** 1099–1103 (2001). 10.1038/35074106 11323673

[b23] Boring L., Gosling J., Cleary M. & Charo I. F. Decreased lesion formation in CCR2-/- mice reveals a role for chemokines in the initiation of atherosclerosis. Nature 394, 894–897 (1998). 9732872 10.1038/29788

[b24] Michelsen K. S. . Lack of Toll-like receptor 4 or myeloid differentiation factor 88 reduces atherosclerosis and alters plaque phenotype in mice deficient in apolipoprotein E. Proc Natl Acad Sci USA 101, 10679–10684 (2004). 15249654 10.1073/pnas.0403249101 PMC489994

[b25] Gu L. . Absence of monocyte chemoattractant protein-1 reduces atherosclerosis in low density lipoprotein receptor-deficient mice. Mol Cell 2, 275–281 (1998). 9734366 10.1016/s1097-2765(00)80139-2

[b26] Crellin N. K. . Human CD4 + T cells express TLR5 and its ligand flagellin enhances the suppressive capacity and expression of FOXP3 in CD4 + CD25 + T regulatory cells. J Immunol 175, 8051–8059 (2005). 16339542 10.4049/jimmunol.175.12.8051

[b27] Hasu M., Thabet M., Tam N. & Whitman S. C. Specific loss of toll-like receptor 2 on bone marrow derived cells decreases atherosclerosis in LDL receptor null mice. Can J Physiol Pharmacol 89, 737–42 (2011). 21895526 10.1139/y11-071

