# Supplementary material for: Leukocyte TLR5 deficiency inhibits atherosclerosis by reduced macrophage recruitment and defective T-cell responsiveness
Source: Sci Rep. 2017 Feb 16;7:42688. doi: 10.1038/srep42688 (PMC5311952; doi:10.1038/srep42688)
Supplement: Supplementary Figure [file srep42688-s1.pdf]

Leukocyte TLR5 deficiency inhibits atherosclerosis by reduced macrophage recruitment and defective T-cell responsiveness

Guilielmus H.J.M. Ellenbroek[1], Gijs H.M. van Puijvelde[2], Adam A. Anas[3,4], Martine Bot[2], Miriam Asbach[2], Arjan Schoneveld[1], Peter J. van Santbrink2, Amanda C. Foks[2], Leo Timmers[5], Pieter A. Doevendans[5], Gerard Pasterkamp[1,6], Imo E. Hoefer[1,6], Tom van der Poll[3,4], Johan Kuiper[2], Saskia C.A. de Jager[1,2]

Supplemental figure 1: Gating strategy for monocytes

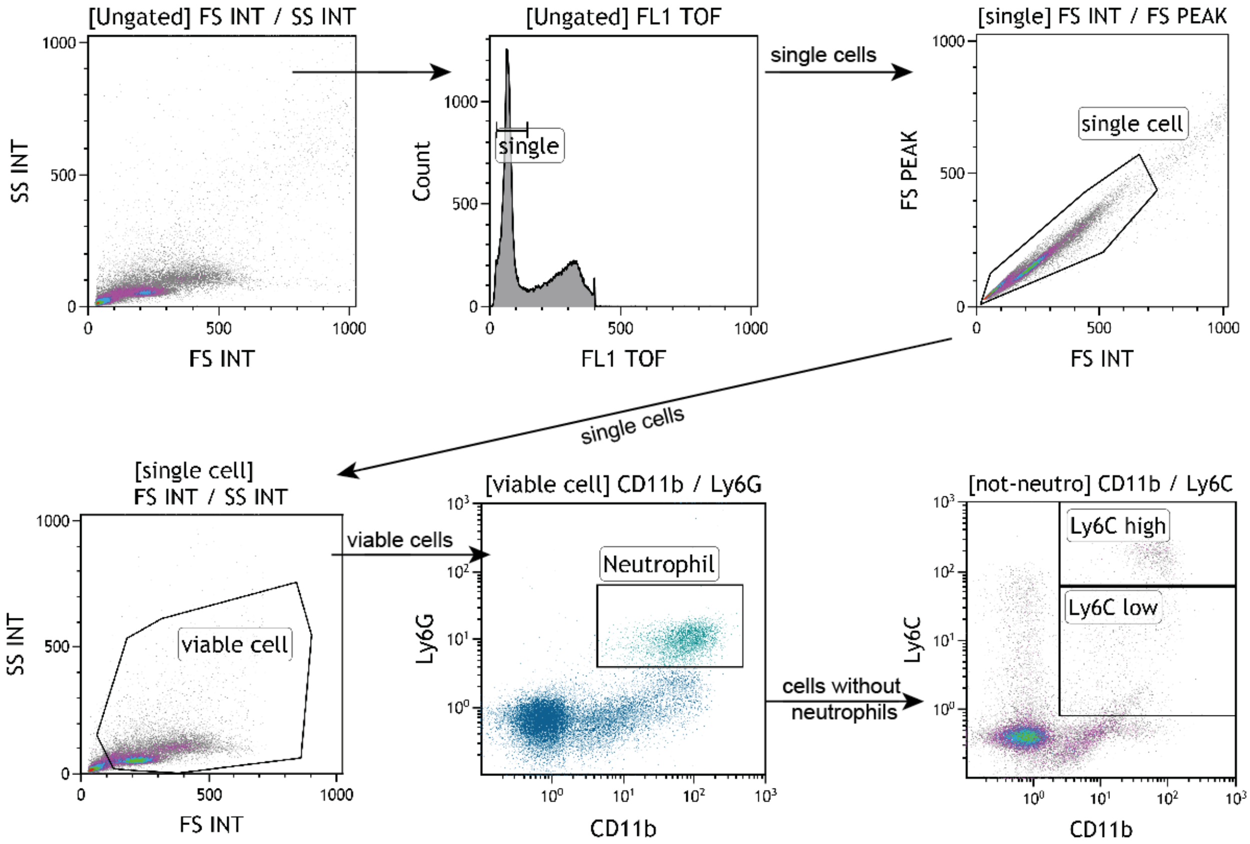

Single cells were selected based on time of flight combined with the integral and peak forward scatter and forward to sideward scatter. Subsequently neutrophils were gated out based on CD11b and Ly6G (1A8) expression. All remaining cells were plotted for CD11b and Ly6C after which we were able to gate Ly6C high and Ly6C low monocytes.
